# Cycling around a Curve: The Effect of Cycling Speed on Steering and Gaze Behavior

**DOI:** 10.1371/journal.pone.0102792

**Published:** 2014-07-28

**Authors:** Pieter Vansteenkiste, David Van Hamme, Peter Veelaert, Renaat Philippaerts, Greet Cardon, Matthieu Lenoir

**Affiliations:** 1 Department of Movement and Sports Sciences, Ghent University, Ghent, Belgium; 2 Department of Telecommunications and Information Processing, Ghent University, Ghent, Belgium; University of Muenster, Germany

## Abstract

Although it is generally accepted that visual information guides steering, it is still unclear whether a curvature matching strategy or a ‘look where you are going’ strategy is used while steering through a curved road. The current experiment investigated to what extent the existing models for curve driving also apply to cycling around a curve, and tested the influence of cycling speed on steering and gaze behavior. Twenty-five participants were asked to cycle through a semicircular lane three consecutive times at three different speeds while staying in the center of the lane. The observed steering behavior suggests that an anticipatory steering strategy was used at curve entrance and a compensatory strategy was used to steer through the actual bend of the curve. A shift of gaze from the center to the inside edge of the lane indicates that at low cycling speed, the ‘look where you are going’ strategy was preferred, while at higher cycling speeds participants seemed to prefer the curvature matching strategy. Authors suggest that visual information from both steering strategies contributes to the steering system and can be used in a flexible way. Based on a familiarization effect, it can be assumed that steering is not only guided by vision but that a short-term learning component should also be taken into account.

## Introduction

The role of eye movements in curve negotiation has been the subject of research for more than 35 years. Although it is generally accepted that visual information guides steering [1]–[5], there is no consensus on how gaze behavior contributes to steering through curves.

In their well-known experiment, Land & Horwood [6] showed that at higher speeds (>12 m/s) car drivers look at the road more than 1 s ahead to gain information about its curvature, while position-in-lane information is obtained from the nearer part of the road approximately 0.5 s ahead. Although there has been some discussion about the size and location of these two regions [7], [8], it is generally accepted that both road curvature information and position-in-lane information are needed for efficient curve negotiation. Since position-in-lane information can be gathered using ambient vision, fixations are mainly directed to the far region. However, the exact location of drivers' gaze and its influence on steering corrections remains a debated issue.

**Figure 1:**
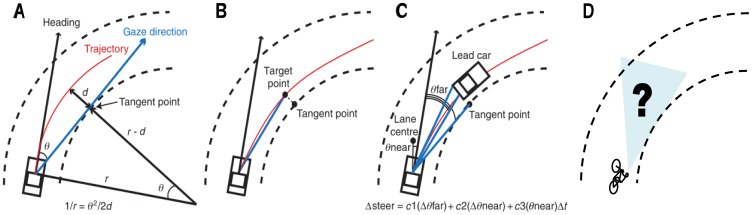
Steering models for car driving (A–C) and cycling (D). (A) tangent point strategy according to Land and Lee (1994), (B) ‘look where you want to go’ strategy, (C) two-point visual control model of Salvucci and Gray (2004), and (D) visual behavior while cycling through curves has never been studied (adapted from Mars 2008).

With respect to curve negotiation, a possible source of road curvature information is the ‘tangent point’ [2]. This is the innermost point of a curve from the driver's point of view, and its direction relative to the current heading of the vehicle is a good predictor of the road curvature (see Figure 1). Since the gaze angle towards the tangent point and the steering wheel angle are very similar, the tangent point can be used as a pursuit control signal for steering [9]. Pursuit control implies that observed characteristics of a previewed track are transformed directly into steering commands in a continuous fashion. In this case, changes in the visual direction of the tangent point will result in corresponding changes in the steering angle. Therefore, the tangent point has been put forward as an ideal reference point to estimate road curvature and to maintain a trajectory at a fixed distance from the inside edge [10], [11]. This strategy of steering through a curve has been referred to as the tangent point strategy. However, Tresilian [9] argued that the use of this particular steering strategy is not absolutely necessary for successful curve negotiation. Other points on the inner edge of a curve could also serve as pursuit control signal and, therefore, guide steering. Furthermore, many studies report the occurrence of gaze near the tangent point, not necessarily at the tangent point itself. Given that this steering strategy uses visual information from the inside edge of the curve to maintain a trajectory at a fixed distance from the inside lane, the current article will refer to the tangent point strategy as the curvature matching strategy from this point onwards.

Several studies have confirmed that the inside edge close to the tangent point is often gazed at during curve negotiation [11]–[13]. However, Wilkie et al. [5] pointed out a number of problems associated with the use of the curvature matching strategy. A first issue is that this strategy only applies to bends with a continuous inside curb or edge line. Therefore, it is questionable whether the curvature matching strategy can be generalized to all types of roads. Furthermore, the studies favoring the curvature matching strategy did not instruct the car drivers about the road position they should maintain. Because of the natural tendency to ‘cut the corner’ [14], the drivers might just have been watching where they were going. When the drivers were asked to keep their car in the center or the outside of the lane, it was found that gaze is mainly directed to points on the future path [15]. This observation of Kountouriotis et al. [15] suggests that when steering towards the inside edge of a bend, looking to the inside edge (e.g., the tangent point) could be caused by a ‘look where you are going’ strategy rather than a curvature matching strategy [5].

According to the ‘look where you are going’ strategy (which has also been referred to as ‘viapoint strategy’ and ‘future path strategy’), drivers look at a point through which they will actually pass 1–2 seconds ahead of their current position [10]. When negotiating a curve and looking at a point on the desired future path, a combination of information from retinal flow, gaze angle and rate of rotation relative to gaze position provides visual signals about whether the steering angle needs to be remained, increased or decreased [16], [17]. This ‘look where you are going’ strategy is in line with several studies using a wide range of experimental set-ups to confirm that gaze is usually directed in the direction of traveling [18]–[22]. Due to the large variation of experimental set-ups that have been used to test gaze behavior during locomotion, there is also a considerable variation in the gaze distribution reported in several studies. Since gaze behavior is very task and environment dependent [23]–[24], differences in speed, visibility, curvature type (open vs. closed), curvature radius, imposed task (none or stay central) and location (real road vs. simulator) may have caused this variation in literature. In addition, different measurements of gaze and steering behavior have been used, which complicates the comparison of study outcomes. Nevertheless, this diversity in experimental set-ups helps to develop a more general theory for gaze behavior during locomotion. Given that recent studies suggest a flexible / weighted system for gaze distribution [7], [8], [15], [25], [26], comparing gaze behavior changes under various environmental constraints could lead to more generally applicable models for gaze behavior during locomotion.

Unfortunately, experiments on visual behavior during curve negotiation mainly investigated car driving situations at a single velocity. Since gaze behavior changes according to the traveling speed [25] and might be subject to the type of vehicle that is used, the aim of current study was to explore gaze and steering behavior of cyclists when negotiating a curve at multiple speeds.

Compared to the amount of research conducted in car driving, the transferability of the existing models towards curve cycling is poorly documented. Both vehicles allow faster locomotion than travelling by foot and require steering through a curve to change direction, whereas one can make a point turn when walking and running [27]. However there are many important differences between car driving and cycling that might induce different visual requirements to control locomotion [28]. In a car, the horizontal view is almost unrestricted, but the vertical field of view is restricted by the design of the car (e.g., height of the windshield). As a consequence, the nearest part of the road visible for a car driver is a few meters in front of the driver. A cyclist, on the other hand, has an unrestricted view both in the horizontal as in the vertical plane. This means that the ‘near region’, which provides compensatory closed-loop information, extends to below the cyclist and therefore might provide more feedback from edge lines and visual flow [29]. Furthermore, traveling speed by car is usually much higher than by bike. This will most likely cause cyclists to direct their gaze closer than in car driving experiments [6], [25]. Finally, cyclists also have to maintain balance on their bicycle while cars are stable on their own [30]. Since vision contributes to balance control [31], [32], a part of the visual attention of cyclists might be used to support this. Due to the differences in field of view [28], traveling speed [25] and balance requirements [30], we expect cyclists to have a slightly different gaze behavior than car drivers. Nonetheless, we also expect cyclists to use a curvature matching strategy and/or a ‘look where you are going’ strategy to steer through a curve.

## Methods

### Participants

A convenience sample of twenty-five participants (aged 21.40±0.58 years; 11 females) were recruited from Ghent University students to participate in the experiment. All participants had normal or corrected-to-normal vision and used their bicycle on regular basis for transportation. To ensure reliable eye-tracking data, only data of participants with a tracking ratio above 90% and good pre-post calibration were retained for analysis. Seventeen participants (aged 21.35±0.49 y; 8 females) met these inclusion criteria.

### Apparatus

Gaze was recorded using the Head-mounted Eye-tracking Device (iViewX HED System) and iView X software of SMI (Teltow, GER). The system recorded eye movements of the left eye with a 50 Hz infra-red sensitive camera (using dark pupil position and corneal reflection) and a scene video with a horizontal and vertical field of view of approximately 33° with a 25 Hz camera. Both cameras were mounted on a baseball cap and connected to a notebook (Lenovo 84×201; 1.4 kg) which was worn in a backpack. The system was calibrated using a five-point calibration and has an accuracy of 1° [33].

A 50 Hz HD camera (Panasonic HC-X900) was mounted at the back of the bicycle and pointed backwards to record steering behavior. A full HD digital camera (25 Hz; Panasonic HDC-HS80) was used as an overview camera to record the experiment.

### Experimental setup and procedure

**Figure 2:**
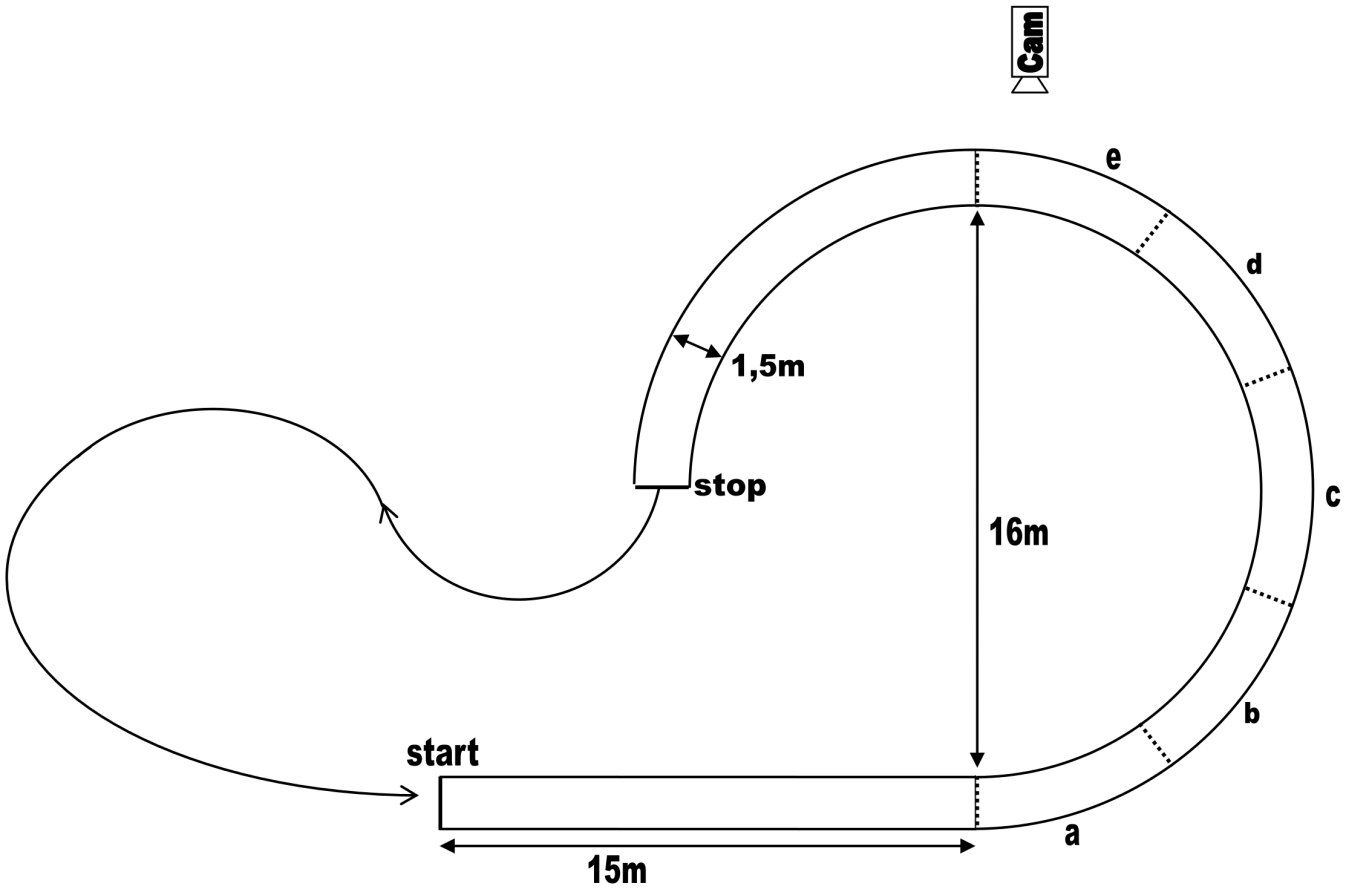
Experimental set-up. Length of the semicircle was 26,3(measured in center of the lane). Dashed lines were not physically present during the experiment but indicate the five segments of the curve (a-e).

In a gymnasium, a 1.5 m wide cycling track was marked on the floor with 2.5 cm wide white tape. The track consisted of a 15 m run-up and a 3/4 circle with a diameter of 16 m (see Figure 2). Two lines marked the start and the end of a semicircle, the remaining 1/4 of the circle served as a buffer so that ‘exit behavior’ only occurred past the semicircle.

On arrival, the participants were briefed about the experiment and were asked to read and sign the informed consent. Both the study and the informed consent were approved by the Ethical Committee of Ghent University Hospital (approval number: OG017). The saddle of an instrumented city bicycle (women's model) was adjusted so that the participants could reach the ground with the tips of their feet while seated. They were then asked to cycle the track at a low (±8 km/h), medium (±14 km/h) and high speed (±19 km/h), corresponding with completing the semicircle in 12.0, 6.7 and 4.9 seconds, respectively. These three speed conditions will be referred to as ‘slow’, ‘medium’ and ‘fast’. During the familiarization trials, particpants' lap time was recorded with a stopwatch and, if necessary, they were instructed to cycle faster or slower. Each speed condition was repeated until the participant managed to cycle the trajectory in the corresponding lap time ±1 second. This usually took only two familiarization trials.

When the participants were familiar with the track and the three speeds, they were asked to put on the eye tracker and secure it with a strap. After calibration the notebook was put in the backpack and the participant was asked to mount the bicycle and line up at the starting line. Participants were asked to ride three consecutive trials through the experimental cycling track at each of the three speeds, which were randomized for each participant. After each speed condition a calibration check was performed.

One of the problems in comparing curvature matching strategies with ‘look where you are going’ strategies, is that both strategies lead to similar gaze behavior when drivers cut into the bend (i.e., gaze to the inner edge) [5], [34]. To ensure that the two strategies would evoke a distinguishable gaze behavior, the participants in current experiment were asked to stay in the center of the track as much as possible. This way, the curvature matching strategy evokes gaze to the inside edge, while using the ‘look where you are going’ strategy evokes gaze to the center of the lane.

### Data analysis

#### Steering behavior

Based on the video images of the bicycle-mounted camera, the cycled trajectory was reconstructed for all 25 participants using the robust visual odometry method of Van Hamme et al. [35]. This method allows for the reconstruction of relative motion with a typical translational accuracy of 0.10% (i.e., longitudinal accuracy) and rotational accuracy of 0.46°/m over a 10 m segment. Manual lateral measurements at the start, middle and end of the semicircle were used to obtain absolute position and to eliminate rotational drift. This method resulted in 100 XY-coordinates per trial and for each of these coordinates, the lateral distance towards the inner edge was calculated.

To obtain a more detailed view on the steering behavior throughout the trial, the semicircle was divided into five segments of 36° each (a–e in Figure 2). For each of the five segments of the semicircle, the lateral distances towards the inner edge were used to calculate *mean lateral deviation from the inner edge (M Lat Dev)* and *standard deviation of lateral deviation from inner edge (SD Lat Dev).* This standard deviation is a measure of how much variation around the average lateral distance each cyclist showed. However, this does not indicate the number of steering corrections. To this end, the number of times that the lateral deviation from the inner edge changed from increasing to decreasing, or vice versa, was counted and divided by the duration of the trial. Accordingly, the *number of steering reversals per second (#SR/s)* was calculated for the total semicircle as well as per segment for each participant.

To verify that the participants did not correct their trajectory by varying their velocity along the semicircle, the *mean velocity per segment* of each participant was extracted by the visual odometry method. To eliminate measurement noise, the obtained velocities were filtered by a type I linear phase lowpass filter with −6 dB amplitude gain at 0.25 Hz.

#### Gaze behavior

Gaze behavior was analyzed by calculating the *dwell time percentage* to specific Areas Of Interest (AOIs). This dwell time percentage is the time spent watching a specific AOI (i.e., the sum of all fixations and saccades that hit the AOI [33]), relative to the duration of the trial (time to complete the semicircle). Dwell time % was calculated using the fixation-by-fixation analysis as described in [36]. For this analysis, fixations were determined by the ‘*SMI fixation detection algorithm*’ in BeGaze 3.3 (SMI, Teltow GER) and superimposed on the scene video. Using the ‘Semantic Gaze Mapping function’ of BeGaze, the fixations shown in this gaze-overlay video were analyzed one-by-one and manually assigned to one of the AOIs by the experimenters. Although fixation location and duration is calculated based on screen coordinates, this method has been described to be a valid and time-saving alternative to the classic frame-by-frame analysis to calculate overall dwell time % to AOIs [36].

**Figure 3:**
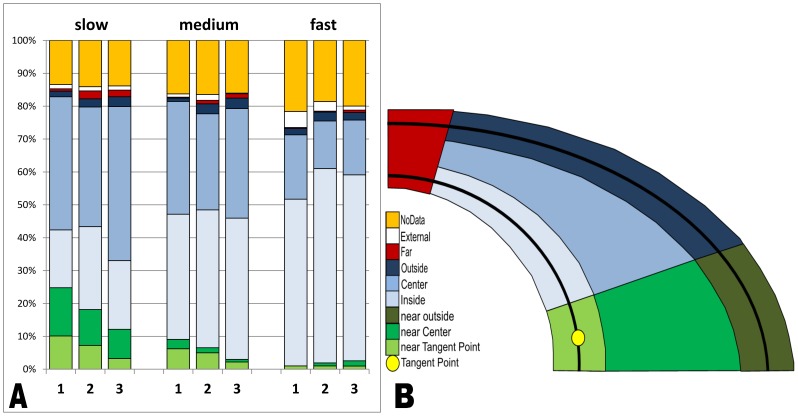
Areas Of Interest and Dwell time percentages. A) Dwell time percentage for each AOI per Trial and Speed, B) A sketch of the AOIs as defined on the gaze overlay videos. Black lines represent the cycling lane. Note that this figure is a sketch of the AOIs, this grid was not used for gaze analysis. Each of the fixations was assigned manually to one of these AOIs as described in the method section.

Gaze location was categorized on two levels: ‘lateral direction’ and ‘depth’. On the ‘lateral’ level, fixations were judged to be either directed towards the ‘*inside edge’*, the ‘*center*’ or the ‘*outside edge*’. On the ‘depth’ level, a distinction was made for fixations that were directed ‘*near*’ (up to approximately 4 m in front of the participant), ‘*middle*’ or ‘*far*’ (looking more than 1/4 of the bend ahead). For the ‘*far*’ fixations however, it was difficult to distinguish between fixations to the inside edge, center or outside edge. Therefore, far fixations were not categorized according to lateral direction. In that way, all fixations to the cycling lane could be categorized to one of the following seven AOIs: ‘near inside’, ‘near center’, ‘near outside’, ‘inside’, ‘center’, ‘outside’ and ‘far’. A sketch of how the AOIs were spread across the scene video can be found in Figure 3B.

Considering that the participants in the current experiment were instructed to cycle in the center of the lane, the location of the tangent point was approximately 3.7 m ahead of the cyclists. Therefore, fixations towards the tangent point (the innermost point of the curve from the cyclist's point of view) were labeled under ‘near inside’. All fixations that fell outside of one of the previous AOIs were assigned to the category ‘other’. The difference between 100% and the sum of the eight AOIs was called ‘NoData’ and represents saccades between AOIs, blinks and data loss during the experiment.

## Results

**Figure 4:**
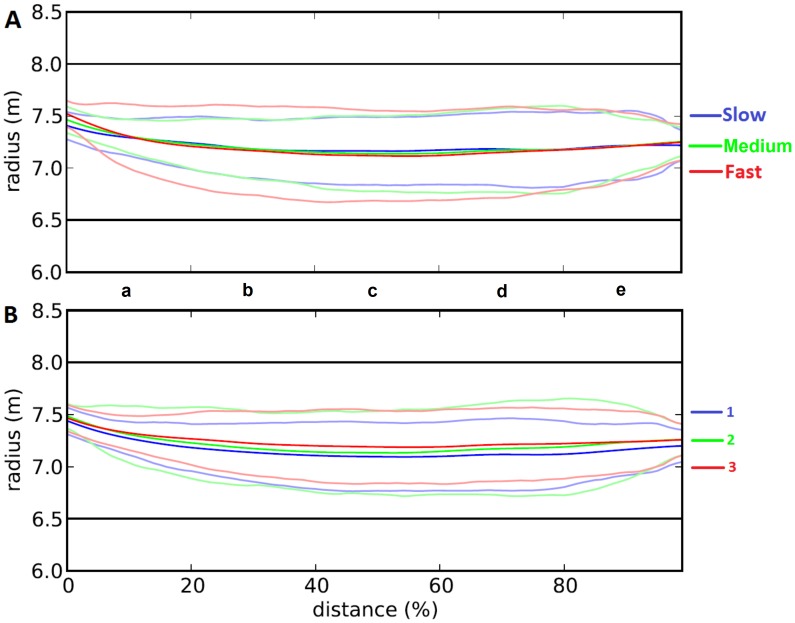
Average cycling trajectory and standard deviation per speed (A) and per trial (B). Straight black lines represent edges of the cycling path. Light colors indicate standard deviation.

All variables were analyzed in SPSS22 using repeated measures ANOVA with the Huynh-feldt correction. Post hoc tests were performed using the Bonferroni correction for pairwise comparison. Significance level for all tests was set at p<0.05. A plot of the average cycling trajectory and standard deviation per speed (A) and per trial (B) can be found in Figure 4.

### Lateral position at curve entrance

The manual measurement of the lateral deviation from the inside edge at the start (Lat Dev Start) was compared across speed conditions and trials to analyze how participants entered the semicircle. Table 1 shows this lateral deviation at the start of the curve per trial for each speed condition. A repeated measures ANOVA with speed and trial as within-subjects factors revealed that participants entered the curve more towards the middle of the lane in the slow condition than in the medium and fast condition (F_2,32_ = 8.402; p = 0.001). Although a significant within-subjects effect was found for trial (F_2,32_ = 4.104; p = 0.026), no significant differences among the trials were found in the pairwise comparisons. However, the analysis also revealed an interaction effect between speed and trial (F_4,60_ = 2.837; p = 0.032) which shows that in the fast condition, participants entered the curve closer to the outside edge in the second and third trial as compared to their first trial. No differences between trials were found at slow and medium speed.

**Table 1:** Average and SD of lateral deviation from inside edge at curve entrance as a function of speed and trial.

Lat Dev Start	Trial 1	Trial 2	Trial 3	Average
**Slow**	0,91±0,15	0,92±0,12^e,f^	0,91±0,13^g^	**0,91±0,13^a,b^**
**Medium**	0,94±0,10	1,01±0,14^e^	0,97±0,10^h^	**0,97±0,12^a^**
**Fast**	0,93±0,12^c,d^	1,02±0,10^c,f^	1,04±0,11^d,g,h^	**1,00±0,12^b^**
**Average**	**0,93±0,12**	**0,98±0,13**	**0,97±0,12**	

Middle of the lane is at 0.725 m from inside edge. Significant differences (p<0.05) are indicated by identical superscript letters.

### Cycling speed

A repeated measures ANOVA with speed condition, trial and segment of the semicircle as within-subjects factors was used to analyze cycling speed. Average cycling speeds per speed condition, trial and segment can be found in Table 2.

**Table 2:** Average and SD of cycling speed in km/h as a function of speed, trial and segment of the semicircle.

	Segment a	Segment b	Segment c	Segment d	Segment e	Average
Slow	8,57±1,30^c,d,e^	8,39±1,16^f^	8,25±1,15^c,f^	8,25±1,15^d^	8,21±1,15^e^	**8,33±1,18^a^**
Medium	13,82±1,33	13,85±1,39^g,h,i^	13,69±1,37^g,j^	13,65±1,38^h,k^	13,53±1,45^i,j,k^	**13,71±1,38^a^**
Fast	18,78±1,07^l^	19,22±1,19^l^	19,16±1,28	19,19±1,29	19,18±1,28	**19,11±1,23^a^**
Trial 1	13,2±4,28^m,t,u^	13,49±4,56^m,v^	13,48±4,61^x^	13,53±4,65	13,49±4,68	**13,44±4,53^b^**
Trial 2	13,86±4,36^t^	13,82±4,63^n,w^	13,70±4,70^Y^	13,73±4,73	13,67±4,73^n^	**13,76±4,60^b^**
Trial 3	14,11±4,47^u^	14,15±4,70^o,p,q,v,w^	13,93±4,71^o,r,s,x,y^	13,83±4,67^p,r^	13,76±4,69^q,s^	**13,96±4,62^b^**
**Average**	**13,72±4,36**	**13,82±4,61**	**13,70±4,64**	**13,70±4,66**	**13,64±4,67**	

Significant differences (p<0.05) are indicated by identical superscript letters. Significant differences between speed conditions across the five segments (average) were also significantly different for each segment.

As instructed, participants cycled slowest in the slow condition and fastest in the fast condition (F_2,32_ = 636.257; p<0.001). They were also found to cycle slightly faster as they repeated the trials (F_2,32_ = 10.559; p<0.002). Although no general differences across segments were observed (F_4,64_ = 2.133; p = 0.157), significant interaction effects between speed and segment (F_8,128_ = 8.319; p<0.001) and between trial and segment (F_8,128_ = 14.478; p<0.001) suggest that in some conditions cycling speed was different between the five segments of the curve. Post hoc results for both interaction effects can also be found in Table 2. These results reveal that there are only minor differences between the three speed conditions in how cycling speed changes over the five segments of the semicircle. The differences in cycling speed between the three consecutive trials are mainly due to differences in the first three segments. In the final two segments of the curve, no significant differences across trials were observed.

### Steering

Steering measures were also analyzed using repeated measures ANOVA with speed condition, trial and segment as within-subjects factors. The results per speed condition and trial can be found in Table 3, whereas averages per segment and the result of pairwise comparison can be found in Table 4.

**Table 3:** Average and SD of steering behavior measures as a function of speed and trial.

	Slow	Medium	Fast	Trial 1	Trial 2	Trial 3
**M Lat Dev (m)**	0,70±0,14	0,70±0,14	0,70±0,17	0,64±0,14^a,b^	0,72±0,15^a^	0,74±0,15^b^
**#SR/s**	0,50±0,49	0,56±0,68	0,64±0,86	0,58±0,71	0,54±0,67	0,59±0,70
**SD Lat Pos**	0,04±0,02^c^	0,04±0,03^d^	0,05±0,04^c,d^	0,04±0,03	0,04±0,03^e^	0,04±0,03^e^

Lat Dev of 0.725 m is center of lane. Significant differences (p<0.05) are indicated by identical superscript letters.

**Table 4:** Average, SD and results of pairwise comparison of steering behavior measures per segment of the semicircle (a–e).

	Segment a	Segment b	Segment c	Segment d	Segment e
**mean lateral deviation (m)**	**0,82±0,11**	**0,68±0,13**	**0,63±0,14**	**0,66±0,16**	**0,70±0,15**
	Segment a	<0.001	<0.001	<0.001	<0.001
	Segment b		<0.001	0.898	1.000
	Segment c			0.109	0.001
	Segment d				<0.001
**#SR/s**	**0,19±0,44**	**0,59±0,74**	**0,70±0,64**	**0,71±0,72**	**0,64±0,76**
	Segment a	<0.001	<0.001	<0.001	<0.001
	Segment b		1.000	1.000	1.000
	Segment c			1.000	1.000
	Segment d				1.000
**SD of lateral deviation**	**0,07±0,04**	**0,03±0,02**	**0,02±0,02**	**0,03±0,02**	**0,04±0,03**
	Segment a	<0.001	<0.001	<0.001	0.001
	Segment b		<0.015	0.173	0.472
	Segment c			1.000	0.001
	Segment d				<0.001

The mean lateral deviation from the inner edge of the semicircle (M Lat Dev) was not affected by cycling speed (F_2,32_ = 0.010; p = 0.989). The analysis per trial (F_2,32_ = 35.380; p<0.001) revealed that the mean lateral deviation was significantly lower in the first trial than in the two subsequent trails. Regardless of the speed condition, significant differences between the five segments of the curve (F_4,64_ = 46.641; p<0.001) show that participants cycled more towards the outside edge in the first segment (a), and more towards the inside edge in the subsequent segments (b–e).

The analysis of the number of steering reversals per second (#SR/s) revealed significantly less corrections in the first segment as compared to the rest of the curve (F_4,64_ = 13.022; p<0.001). No significant effects of speed condition (F_2,32_ = 1.788; p = 0.185) or trial number (F_2,32_ = 0.301; p = 0.735) were found.

The analysis of the standard deviation of lateral deviation from inside edge (SD Lat Dev) indicated significant differences between speed conditions (F_2,32_ = 8.144; p = 0.001), between trials (F_2,32_ = 4.278; p = 0.023) as well as between segments (F_4,64_ = 53.168; p<0.001). Pairwise comparison showed that the SD Lat Dev was higher in the fast condition than in the medium and the slow condition. In addition, SD Lat Dev was lower in the third as compared to the second trial. Results per segment indicate that the largest variations in lateral deviation could be found in the first segment of the curve.

**Figure 5:**
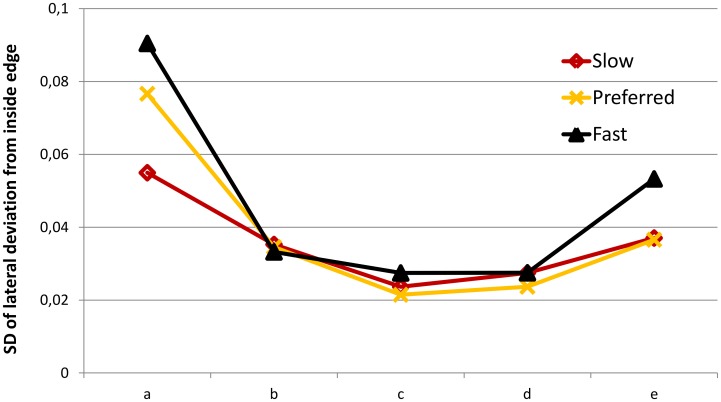
Interaction-effect between speed and Segment on SD of Lateral Deviation. a–e represent the five segments of the curve.

However, the analysis of SD Lat Dev also revealed a significant interaction effect between speed and segment (F_8,128_ = 4.249; p<0.001) and between trial and segment (F_8,128_ = 2.605; p = 0.018). Post hoc results of these interactions can be found in Appendix S1. The most apparent interaction effect is shown in Figure 5 which indicates that the faster the participants cycled, the higher their SD M Lat Dev in the first segment.

### Gaze: Dwell time %

The effects of cycling speed and trial number on dwell time percentages to each area of interest together with the changes throughout the segments of the curve were also analyzed using repeated measures ANOVA with speed condition, trial and segment as within-subjects factors. The results of the dwell time percentages per speed and segment can be found in Table 5. Figure 3A visualizes how gaze was distributed over the AOIs per speed and trial. In general, these results show that gaze was predominantly directed to the inside edge and the central region of the curve. However, Table 5 also shows high standard deviations for the dwell time percentages. Since the within-subject variability was two to three times smaller than the between-subject variability, this suggests that there were notable individual differences in where participants directed their gaze at during the experiment.

**Table 5:** Dwell time percentage in each AOI per speed condition.

Dwell time %	Slow	Medium	Fast	Segment a	Segment b	Segment c	Segment d	Segment e
Near Inside	6,87±14,1	4,45±10	0,97±2,44	5,3±11,02	2,52±5,3	2,93±10,38	4,39±8,18	5,37±9,86
Near Center*	11,48±14,75^a,b^	1,74±2,91^a^	0,83±2,83^b^	6,16±7,25	4,73±5,92	3,65±5,73	4,23±6,16	4,67±6,21
Near Outside	0±0	0±0	0±0	0±0	0±0	0±0	0±0	0±0
Inside*	21,23±21,55^c,d^	40,99±25,83^c^	55,48±27,62^d^	36,23±20,94	39,34±27,47	44,76±25,52	42,28±19,07^e^	33,56±14,24^e^
Center*	41,26±25,53^f^	32,29±24,8^g^	16,91±19,87^f,g^	32,95±20,59^h^	36,95±23,49^i^	32,67±24,53^j^	29,12±18,9^k^	19,07±14,39^h,i,j,k^
Outside	2,37±5,79	2,41±6,21	2,29±6,83	2,08±3,99	2,76±5,02	2,03±3,99	1,94±3,61	2,96±7,28
Far	1,72±5,55	0,89±2,82	0,46±1,15	0,7±2,1	0,82±3,4	1,12±4,61	1,27±5,23	1,19±2,27
External	1,3±1,39	1±1,53	3,01±4,88	2,18±4,21	0,68±1,01	1,75±3,81	2,46±3,38	1,79±2,59
NoData*	13,76±4,16^l,m^	16,24±4,75^l^	20,05±4,08^m^	14,4±4,72^n^	12,19±5,31^o^	11,1±5,62^p^	14,32±6,77^q^	31,39±5,57^n,o,p,q^

*indicates significant Univariate results; Significant differences (p<0.05) of pairwise comparison are indicated by identical superscript letters.

Cycling speed had a significant effect on the time that participants spent watching the areas ‘Near center’ (F_2,32_ = 8.063; p = 0.011), ‘Inside’ (F_2,32_ = 14.428; p<0.001) and ‘Center’ (F_2,32_ = 8.859; p = 0.001). At low cycling speed, gaze was directed more to the near center of the road and less to the inside edge. At high cycling speed, gaze was directed less to the center of the road. Dwell time % to the other AOIs was not significantly affected by cycling speed (p>0.05).

Between the five segments of the semicircle, significant differences in dwell time % were found for the AOIs ‘Inside’ (F_4,64_ = 3.327; p = 0.022) and ‘Center’ (F_4,64_ = 9.162; p<0.001). Dwell time % to ‘Inside’ was lower in the last segment (e) as compared to the second last segment (d), and dwell time % to the center was lower in the last segment than in the rest of the curve.

Dwell time % to all AOIs did not significantly change with increasing trial number and no interaction effects were found (p>0.05).The percentage of NoData changed with increasing speed (F_2,32_ = 12.161; p<0.001) and along segments (F_4,64_ = 42.517; p<0.001), but not with increasing trial number (F_2,32_ = 0.269; p = 0.766). The percentage of ‘NoData’ was lower at low cycling speed, and a higher percentage of ‘NoData’ was found in the last segment than in the rest of the curve.

## Discussion

The current study explored the visual behavior while cycling in the middle of a semicircular lane, and investigated the effect of cycling speed on steering and gaze behavior. Similar to the findings resulting from car driving experiments, cyclists mainly directed their gaze to the inside edge and the center of the curve. However, current results reveal that at higher cycling speeds, participants direct their gaze further and more towards the inside edge than at lower cycling speeds. Except for cutting more into the bend in the first segment of the curve, no effect of cycling speed on steering behavior was found. Furthermore, the results show that participants cycled more towards the center of the bend as they repeated the trajectory.

### Steering behavior

Similar to steering behavior of car drivers during curve negotiation [37], cyclists in the current experiment entered the curve on the outside of the lane and then cut into the first segment of curve (segment a). This was reflected by a higher mean lateral position, a higher SD of lateral position and a lower frequency of steering corrections in the first segment. After the first segment, steering behavior was characterized by a stable lateral position and more steering corrections. These findings suggest that a different steering strategy is used at curve entrance (segment a) than during the cornering phase (segments b–e) of the semicircle.

At curve entrance, participants seem to minimize the lateral acceleration by choosing a path with a lower maximum curvature. According to Boer [38] this should lead to i) steering to the left/right side of the lane before the start of the curve, ii) steering into the curve before the curve's onset, and iii) approaching the inner lane boundary in the middle of the curve. Although participants' steering behavior of the run-up to the curve was not analyzed, the outside position at curve entrance confirms the first prediction of Boer [38] and the steering results of the first segment confirm that participants steered into the curve (see Figure 4A). Furthermore, the finding that at higher cycling speeds participants enter the curve more towards the outside and cut more into the first segment of the bend is also in line with the suggestion that participants tried to minimize lateral acceleration. With respect to the third prediction of Boer [38], ‘cutting the corner’, as has been described for bends without cornering phase, was prevented by the length of the semicircle, the relatively narrow lanes and the instructed steering behavior [14]. Instead, participants stabilized their position in the middle of the lane during the cornering phase of the curve in line with the specific steering instructions. Therefore, the third prediction of Boer, that participants should have steered close to the inner lane boundary of the curve was not confirmed.

Nevertheless, we observed that the lowest lateral deviation from the inner edge was found in the middle segment of the curve, which confirms that participants preferred steering towards the inner edge of the lane. However, if searching for the path with the minimal lateral acceleration were to be the main steering strategy during the cornering phase, participants would favor steering towards the outward side of the curve, since curvature is slightly lower there. Hence, a steering bias towards the inside edge during the cornering phase is in contrast with the idea that participants tried to take the path with minimal lateral acceleration. Instead, this steering behavior is in line with the suggestion of Wilkie et al. [5], that drivers oversteer to provide a spatial buffer. As follows, possible steering errors or an unexpected increase in curvature would merely lead the vehicle towards the center of the lane rather than immediately to the outside border. It seems that participants minimized lateral acceleration when entering the curve, but a spatial buffer was preferred during the cornering phase instead of a lower lateral acceleration. An alternative way to deal with lateral acceleration would be to adapt travelling speed [39]. In the current investigation however, participants were asked to cycle at a constant speed and the results did not indicate adjustments to cycling speed to cope with lateral acceleration.

The finding that participants entered the curve more towards the outside edge at higher cycling speeds and cut into the first segment of the curve while making few steering corrections suggests that an anticipatory steering strategy was used when entering the curve. If steering would be purely controlled by compensatory closed-loop behavior, there would be no need to steer to the outside edge at higher speeds and a similar number of steering corrections would be made over the entire curve. In the subsequent cornering phase, on the other hand, steering corrections and a stable lateral position suggests that a compensatory steering strategy was used to stay on track. This reinforces the suggestion of Godthelp [40] that at curve entrance, steering is based on anticipatory open-loop control, whereas during the cornering phase, steering is primarily based on compensatory closed-loop control. According to Shinar et al. [1] this finding should also be reflected in gaze behavior since the primary function of the eye movements is to provide preview information during the approach phase and to reinforce the awareness of other cues during the cornering phase. In the current study, however, gaze behavior was only analyzed in the cornering phase of the curve.

### Gaze behavior

In contrast to some car driving experiments [2], [11], [13], dwell time percentages in the current study show that cyclist spent very little time watching the AOI ‘near inside’, in which the tangent point was located. However, in the current experiment, the tangent point was located only 3.7 m in front of the participants. This means that the tangent point only fell within the preferred look ahead distance (1–2 s ahead) in the slow cycling condition. As a consequence the tangent point was probably too close to be eligible as a good source for visual information. Instead of looking at the tangent point, gaze was predominantly directed toward the center and the inside edge of the bend, similar to the results of Kountouriotis et al. [15] and Robertshaw et al. [41]. However, high standard deviations of dwell time percentages show that there were notable individual differences in where participants were looking during the experiment. This is in line with earlier results of gaze behavior during cycling [25], [42] and suggests that individual differences in how vision is used to guide steering exist. Notwithstanding the variation of gaze behavior among the participants, an increase of cycling speed had a similar effect on the visual behavior of all participants. As they were instructed to cycle faster, their gaze was less directed to the near region and shifted from a predominantly central road position towards the inner edge of the lane. Interestingly, this shift of gaze was not accompanied by a steering bias towards the inner edge of the curve.

The anticipatory steering behavior that was revealed in the first segment of the curve was not accompanied by a different gaze behavior. Since gaze is proactive, anticipatory gaze behavior might have taken place in the run-up to the curve, which was not analyzed in current experiment. Gaze behavior per segment did reveal a decrease of looking towards the inside edge and center region in the last segment. However, an increase of ‘NoData’ suggests that this decrease of dwell time percentage was caused by more data loss in the last segment. It is possible that the participants started to anticipate the exit of the curve in the last segment, which may have led to a gaze behavior that was more prone to data loss.

#### Effect of speed on look-ahead distance

It has repeatedly been suggested that, when steering through curves, gaze is mainly directed to the road about 1 to 2 seconds ahead [2], [10], [43]. If a constant gaze-steering span (visual buffer) is used, gaze should be directed further ahead at higher speeds and vice versa. For the current experiment, a gaze-action span of 1 to 2 seconds would mean that gaze would have been directed 2.2–4.5 m ahead in the slow condition, 3.8–7.6 m in the medium, and 5.3–10.6 m in the fast condition. Unfortunately, with the gaze analysis used in the current experiment, it was not possible to calculate the exact look-ahead distance of gaze. Nevertheless, as cycling speed increases, a decreasing percentage of dwell time towards the near region (up to ±3–4 m ahead) was found, reflecting a larger look-ahead distance, which is in line with the idea of a constant temporal size of the gaze-steering span [25].

Alternatively, at lower speeds, gaze could have been directed more to the near region due to the increased need for balance. At lower cycling speeds, bicycles becomes less stable [30] and therefore more steering corrections are necessary to maintain balance. Surprisingly, no effect of speed was found on the number of steering reversals. However, as previously suggested [42], changing visual behavior can be the first step to cope with higher task demands. In the current experiment, increased visual attention towards the near region could have been enough to cope with the higher demand of balance control. Therefore, steering behavior was not (yet) affected.

The lack of an increase in dwell time towards the far area was likely due to the fact that it was located further than 10 m from the participant, and thus beyond the area 1–2 seconds ahead. Therefore, the far region in the current experiment could be compared to the ‘occlusion point’ described by Lehtonen et al. [44] rather than to the far region described by Land & Horwood [6]. Considering its distance from the participant, gaze to the far area would serve as anticipatory open-loop control (guidance level [45]). Given that a familiarized trajectory without obstacles and oncoming traffic was used, there was almost no need for anticipatory glances towards the far area, leading to very few fixations in this area.

It must be taken into account that no exact look-ahead distances were measured in the current experiment. Using a head-mounted eye-tracker without head tracking, it is extremely cumbersome and time-consuming to retrieve actual look-ahead distance. Therefore, the experimenters made an estimate of the look-ahead distance based on reference dimensions in the scenery and categorized the fixations as ‘near’, ‘middle’ (blue AOIs in Fig. 3) or ‘far’. Although less accurate, this method was found effective to distinguish between the three look-ahead categories and gives an overview of gaze distribution. Nevertheless, further experiments should try to develop a method measuring actual gaze distance in real-life settings to further investigate the effect of driving/cycling speed on exact look-ahead distance.

#### Effect of speed on gaze to inner edge

Participants mainly looked at the center of the road when cycling at lower speeds, while gaze shifted to the inside edge of the curve at higher cycling speeds. This switch of visual attention is compatible with a switch from a ‘look where you are going’ strategy to a ‘curvature matching’ strategy. According to Wilkie and Wann [10] “The ‘curvature matching’ strategy provides a solution for maintaining a trajectory at a fixed distance from the inside edge, whereas the ‘look where you are going’ strategy allows any curved path to be chosen”. Although most experiments favor one of both strategies, there is no evidence that these strategies are mutually exclusive [9]. Similar to the weighted way in which near and far road information are used to guide steering [8], [15], visual information from the upcoming road and from the inner lane (e.g., tangent point) are possibly also used in a flexible way. Results of the current experiment are in line with the idea that, according to the quality and availability of the visual cues, both strategies contribute to the steering system. Furthermore, using the ‘look where you are going’ as well as the ‘curvature matching’ strategy in a flexible way would also explain the high standard deviations of dwell time percentages in the current experiment and the variation of gaze direction in most previous experiments involving curve negotiation.

At higher speeds the ‘curvature matching’ strategy was possibly more advantageous than at lower speeds. As a consequence, gaze shifted from the center of the road towards the inner lane. However, the question remains whether a different visual input (visual flow) or a higher task demand (higher lateral acceleration) triggered the shift of gaze strategy at higher cycling speeds.

### Effect of trial on steering and gaze behavior

Kandil et al. [11] showed that gaze behavior while negotiating curves changes with familiarization. However, since in natural steering situations a curve is not repeated several times in succession, we believed that the gaze and steering behavior in the current experiment would resemble natural behavior to a greater extent with only a minimum of familiarization trials. Therefore, participants were given no more familiarization trials than necessary to get used to the required speeds.

When checking for an effect of trial, results indeed showed that gaze did not significantly differ across successive trials. Surprisingly, however, participants were found to cycle more towards the center of the lane as they repeated the trial. Yet, both the curvature matching strategy and the future path strategy rely on visual cues to guide steering. Since these cues did not change across trials, repeating the bend should not result in different gaze or steering behavior. Changing steering behavior over successive trials indicates that the participants did not solely rely on visual cues to guide steering but also on previous experiences.

To date, most models of gaze/steering behavior do not incorporate the influence of road familiarity or other previous experiences except for the steering model of McRuer et al. [46], in which a ‘precognitive control loop’ was active next to a compensatory and a feed forward loop. Although the current results are not in line with an open loop precognitive control mechanism as proposed by McRuer et al. [46], they do reinforce the idea of an additional control level that incorporates a familiarity/learning component that influences the steering, and possibly also the gaze behavior.

### Transferability to real road behavior

Although this was a non-simulated experiment, it does not necessarily reflect actual in-traffic gaze and steering behavior. The current experiment was carried out in a distraction-free environment and included only one curve with a constant radius. Therefore, there was a minimal need for anticipatory gaze behavior. The current investigation also focused on the gaze and steering behavior only after curve entrance, while many of the previous curve driving experiments included both the approach as well as the cornering phase. Therefore, the suggestion that the ‘curvature matching’ strategy and the ‘look where you are going’ strategy are used together in a flexible way should be tested on curves with different radii. Nevertheless, the findings of the current experiment contribute to the general understanding of how visual information guides steering through curves.

## Conclusions

The current experiment was the first of its kind to test the gaze and steering behavior of cyclists while steering through a curve. It reinforced the idea that an open-loop anticipatory steering strategy is used at curve entrance, while a closed-loop compensatory strategy is used to steer through the rest of the curve. The gaze behavior of the cyclists was comparable to gaze behavior previously described for car driving. By testing the effect of cycling speed, we added new insights to the discussion whether a ‘curvature matching’ strategy or a ‘look where you are going’ strategy is used during curve negotiation. It can be argued that the ‘curvature matching’ strategy and the ‘look where you are going’ strategy are not mutually exclusive and that, dependent on task constraints and the availability and quality of the visual cues, visual information from both strategies likely contribute to the steering system. Finally, the familiarization effect observed in the current experiment is assumed to reinforce the idea that steering models should take a learning component into account.

## Supporting Information

Appendix S1 **Speed – segment, and trial - segment interactions of SD Lat Dev.**
(DOCX)
