# Supplementary material for: Cycling around a Curve: The Effect of Cycling Speed on Steering and Gaze Behavior
Source: PLoS One. 2014 Jul 28;9(7):e102792. doi: 10.1371/journal.pone.0102792 (PMC4113223; doi:10.1371/journal.pone.0102792)
Supplement: Appendix S1 — Speed – segment, and trial - segment interactions of SD Lat Dev. (DOCX) [file pone.0102792.s001.docx]

Appendix S1: speed – segment, and trial - segment interactions of SD Lat Dev.

|  | a | b | c | d | e | **Av.** |
| --- | --- | --- | --- | --- | --- | --- |
| Slow | 0,05 ± 0,03^a,b,c,p,q^ | 0,04 ± 0,02^a,d^ | 0,02 ± 0,01^b,d^ | 0,03 ± 0,02^c^ | 0,04 ± 0,03 | **0,04 ± 0,02^a^** |
| Medium | 0,08 ± 0,03^e,f,g,h,p^ | 0,03 ± 0,02^e^ | 0,02 ± 0,02^f,i^ | 0,02 ± 0,02^g^ | 0,04 ± 0,02^h,i^ | **0,04 ± 0,03^b^** |
| Fast | 0,09 ± 0,04^j,k,l,m,q^ | 0,03 ± 0,02^j^ | 0,03 ± 0,02^k,n^ | 0,03 ± 0,02^l,o^ | 0,05 ± 0,04^m,n,o^ | **0,05 ± 0,04^a,b^** |
| 1 | 0,08 ± 0,03^a,b,c,d^ | 0,03 ± 0,02^a,e^ | 0,02 ± 0,02^b,f^ | 0,03 ± 0,02^c,g^ | 0,04 ± 0,03^d,e,f,g^ | **0,04 ± 0,03** |
| 2 | 0,08 ± 0,04^h,i,j,k^ | 0,04 ± 0,02^h,l,m^ | 0,02 ± 0,02^i,l,n^ | 0,03 ± 0,02^j,o^ | 0,05 ± 0,04^k,m,n,o,v^ | **0,04 ± 0,03^c^** |
| 3 | 0,06 ± 0,04^p,q,r,s^ | 0,04 ± 0,02^p,t,u^ | 0,02 ± 0,01^q,t^ | 0,02 ± 0,02^r,u^ | 0,03 ± 0,02^s,v^ | **0,04 ± 0,03^c^** |
| **Av.** | **0,07 ± 0,04^d,e,f,g^** | **0,03 ± 0,02^d,h^** | **0,02 ± 0,02^e,h,j^** | **0,03 ± 0,02^f,i^** | **0,04 ± 0,03^g,i,j^** |  |
